# Epidemiology of Birth Defects Based on a Birth Defect Surveillance System from 2005 to 2014 in Hunan Province, China

**DOI:** 10.1371/journal.pone.0147280

**Published:** 2016-01-26

**Authors:** Donghua Xie, Tubao Yang, Zhiyu Liu, Hua Wang

**Affiliations:** 1 Department of Epidemiology and Health Statistic, School of Public Health, Central South University, 110 Xiangya Road, Changsha, Hunan, 410078, P.R of China; 2 Department of Information management, Maternal and Children hospital of Hunan province, 58 Xiangchun Road, Changsha, Hunan, 410078, P.R of China; Tabriz University of Medical Sciences, ISLAMIC REPUBLIC OF IRAN

## Abstract

**Objective:**

To describe the epidemiology of birth defects (BDs) in perinatal infants in Hunan Province, China, between 2005 and 2014.

**Methods:**

The BD surveillance data of perinatal infants (for stillbirth, dead fetus or live birth between 28 weeks of gestation and 7 days after birth) were collected from 52 registered hospitals of Hunan between 2005 and 2014. The prevalence rates of BDs with 95% confidence interval (CI) and crude odds ratio (ORs) were calculated to examine the associations of infant gender, maternal age, and region (urban vs rural) with BDs.

**Results:**

From 2005 to 2014, there were a total of 925413 perinatal infants of which 17753 had BDs, with the average prevalence of 191.84 per 10000 PIs (perinatal infants), showing a significant uptrend. The risks of BDs are higher in urban areas versus rural areas (OR = 1.20), in male infants versus female infants (OR = 1.19), and in mothers above age 35 versus those below age 35 (OR = 1.24). The main five types of BDs are Congenital heart defects (CHD), Other malformation of external ear (OMEE), Polydactyly, Congenital malformation of kidney (CMK), and Congenital talipes equinovarus (CTE). From 2005 to 2014, the prevalence rates (per 10000 PIs) of CHD and CMK increased significantly from 22.56 to 74 (*OR* = 3.29, 95%CI: 2.65–4.11) and from 7.61 to 14.62 (*OR* = 1.92, 95%CI:1.30–2.84), respectively; the prevalence rates of congenital hydrocephalus and neural tube defects (NTDs) decreased significantly from 11.8 to 5.29 (*OR* = 0.45, 95%CI: 0.31–0.65) and from 7.87 to 1.74 (*OR* = 0.22, 95%CI: 0.13–0.38), respectively.

**Conclusions:**

The prevalence rates of specific BDs in perinatal infants in Hunan have changed in the last decade. Urban pregnant women, male perinatal infants, and mothers above age 35 present different prevalence rates of BDs. Wider use of new diagnosis technology, improving the ability of monitoring, strengthening the publicity and education are important to reduce the prevalence of BDs.

## Introduction

Birth defects (BDs) or congenital anomalies are defined by World Health Organization (WHO) as structural, functional and/or biochemical-molecular defects present at birth whether or not detected at that time[1]. WHO estimates that the total prevalence rates of BDs in the developed, middle-income and low-income countries are 47.2, 55.7 and 64.2 per 1000 live births, respectively. The prevalence rate of BDs in China is about 56.0 per 1000 live births, closer to the level of the middle-income countries[2, 3]. There are 900,000 new cases with BDs each year in China, and about 250,000 of them are born with obvious manifestations[4]. BDs have brought tremendous economic burdens to both society and families, and reduced the average life expectancy and quality of newborns [1, 5].

The causes of BDs are complex and obscure and assumed to be a multi-factor effect involving gene factors, environmental factors or their interaction[6, 7],which highlights the urgency to describe the epidemiology of BDs. The Ministry of Health of China initiated a national Hospital-based Birth Defect Surveillance System in1986. This system was designed to track the perinatal infants including live birth up to 7 days, fetal death or stillbirth after 28 weeks of gestation(including termination of pregnancy due to fetal anomaly[8]). Among 8000–10000 types of BDs identified previously, 23 types of BDs were registered in detail and other types of BDs were classified ‘others’ in this surveillance system[9].

In recent years, Hunan province has been confronted with huge challenges in BDs prevention, as the prevalence rates of BDs in Hunan ranked third in China in 2011, fourth in 2012, and fifth in 2013. The Hunan part of the Surveillance System started in 1991involving 52 hospitals (among which 4 hospitals started in 2003 and 11 hospitals started in 2007) and recorded information such as incidence, distribution, and determinants of BDs. Apart from the 23 types required by the state[4, 10–12],other BDs such as congenital malformation of kidney (CMK) were also reported. In this study, we aim to analyze the temporal trend and epidemiological characteristics of BDs in Hunan from 2005 to2014.

## Methods

### Study population

This study involved all the infants (including stillbirth, dead fetus or live birth) during perinatal period (between 28 weeks of gestation and 7 days after birth) born in the 52 registered hospitals of Hunan between 2005 and 2014. The patient records were anonymized and de-identified prior to analysis. The study was approved by the Medical Ethics Committee of the Maternal and Children Hospital of Hunan.

### Surveillance data

The surveillance data of BDs were collected from the obstetrics departments or neonatal departments according to “Maternal and Child Health Monitoring Manual in China” formulated by the National Health and Family Planning Commission. The information, including a case card of each child with BDs, and a quarterly table with the number of perinatal births for each registered hospital was completed by professional gynecological and pediatric or neonatal doctors. Each case card recorded the maternal information(including her residence, economy, education, illness and drug use during pregnancy), the information of baby birth, diagnosis of BDs, and the family history of diseases. Each quarterly table included three months of data from both urban and rural hospitals, such as number of perinatal infants at each maternal age group, number of stillbirths, number of newborns died within 7 days, and number of BDs. The case card and the quarterly table were reported both in paper and online, and were audited by maternal/child health hospitals and health administrative departments, respectively. Periodical quality controls of the monitored hospitals were inspected and examined once every quarter at county-level and half-yearly at city-level or province-level to reduce misstatement or failing to report.

### Criteria of BDs diagnosis

The diagnosis of BDs was based on the Chinese National Criteria of Birth Defects and Tiny Deformities stated in the Manual above and was confirmed after birth(postural defects are excluded in the monitoring system). The diagnosis covered 24 types of BDs and others as a whole (malformation of kidney is separated from others). The diagnosis was made by the departments of B-ultra room, medical genetics, obstetrics, pediatrics, pathology, or clinical laboratory. Experts from each monitored hospital were in charge of diagnostic confirmation and provided technical support for diagnosis. The complex BDs were diagnosed in a higher-level hospital or with expert consultation.

### Statistical analysis

The prevalence rates and 95% confidence intervals (CI) of BDs were calculated both across all years and for each year separately. Chi-squred test was performed to identify the changing trends of prevalence rates of total BDs by year. The prevalence rates of 24 types of BDs were also calculated separatelyand ranked in a descending order. Crude odds ratios (*ORs*) were computed to examine the association of each maternal characteristic with BDs. All analyses were performed on SPSS 18.0(SPSS, Chicago, IL), with significance level at *P*≤0.05.

## Results

### Prevalence and trend of total BDs

From 2005 to 2014, a total of 925413 perinatal infants(about12.33% of total births in Hunan, 925413/7507635) were monitored and17753 babies were found with BDs, with the average prevalence of 191.84 (95%CI:191.04–192.64) per 10000 PIs (perinatal infants). Trend analysis shows that the annual prevalence rates of total BDs in these 10 years increased linearly (*x*^2^trend = 201.361,*P* = 0.000). The prevalence rate of total BDs in Hunan increased by 3.97% annually from 2005 to 2014 (Table 1).

**Table 1:** Prevalence rates of total BDs in Hunan from 2005 to 2014.

year	Perinatal infants	BDs	Prevalence of BDs per 10000 PIs (95%CI)	Fixed base growth rate(%)
2005	38121	573	150.31(146.70–153.90)	-
2006	41559	690	166.03(162.45–169.55)	10.46
2007	69436	1045	150.50(147.85–153.15)	0.13
2008	78376	1346	171.74(169.09–174.39)	14.26
2009	86929	1470	169.10(166.60–171.60)	12.50
2010	98624	1847	187.28(184.28–190.28)	24.60
2011	107500	2449	227.81(225.31–230.31)	51.56
2012	125583	2574	204.96(202.76–207.16)	36.36
2013	135645	2572	189.61(187.51–191.71)	26.15
2014	143640	3187	221.87(219.72–224.02)	47.61
Total	925413	17753	191.84(191.04–192.64)	-

### Prevalence rates and trends of 24 types of BDs

From 2005 to 2014, the main five types of BDs are congenital heart defects(CHD),other malformation of external ear(OMEE), polydactyly, CMK and congenital talipes equinovarus(CTE), with prevalence rates of 53.1,17.39, 15.43, 11.7 and 9.05 per 10000PIs, respectively (Table 2). From 2005 to 2014, the prevalence rates(per 10000 PIs) of CHD, CMK, polydactyly, OMEE, and CTE significantly increased by 51.44 from 22.56 to 74 (*OR* = 3.29, 95%CI: 2.65–4.11,*P* = 0.000), by 7.01 from 7.61 to 14.62(*OR* = 1.92, 95%CI: 1.30–2.84, *P* = 0.001), by 5.23 from 12.59 to 17.82 (*OR* = 1.42, 95%CI: 1.04–1.93, *P* = 0.026), by 2.36 from15.74 to18.1 (*OR* = 1.18, 95%CI: 0.89–1.56, *P* = 0.246),and by 1.95 from 6.82 to8.77 (OR = 1.29, 95%CI: 0.84–1.96, *P* = 0.241), respectively. However, the prevalence rates(per 10000 PIs) of congenital hydrocephalus, neural tube defects (NTDs, including anencephalia, spina bifida, and encephalocele), cleft lip with cleft palate, cleft lip without cleft palate, and limb reduction defects significantly decreased by 6.51 from11.8 to 5.29 (*OR* = 0.45, 95%CI: 0.31–0.65, *P* = 0.000), by 6.13 from 7.87 to 1.74 (*OR* = 0.22, 95%CI: 0.13–0.38, *P* = 0.000),by 4.64 from 9.44 to 4.8 (*OR* = 0.51, 95%CI: 0.34–0.76, *P* = 0.000),by 3.1 from6.3 to 3.2 (OR = 0.51, 95%CI: 0.31–0.83, *P* = 0.006), and by 2.85 from 6.82 to 3.97 (*OR* = 0.58, 95%CI: 0.37–0.93, *P* = 0.020), respectively.

**Table 2:** Prevalence rates of 24 types of BDs in Hunan from2005 to 2014(per 10000 PIs).

Types of BDs	2005	2006	2007	2008	2009	2010	2011	2012	2013	2014	total	rank	D_♀_	Extent%∆	P
Neural tube defects	7.87	5.53	6.05	5.36	5.64	4.66	2.79	2.07	2.65	1.74	3.77	—	-6.13	-77.89	**
Anencephaly	3.93	1.68	2.45	2.3	2.19	1.01	0.84	0.4	0.88	0.42	1.28	17	-3.51	-89.31	**
Spina bifida	2.62	2.17	2.59	2.68	2.07	1.93	1.77	1.04	1.33	1.11	1.74	15	-1.51	-57.63	**
Encephalocele	1.31	1.68	1.01	0.38	1.38	1.72	0.19	0.64	0.44	0.21	0.76	21	-1.1	-83.97	**
Congenital hydrocephalus	11.8	10.11	6.91	6.38	5.98	5.98	5.02	4.3	4.5	5.29	5.85	7	-6.51	-55.17	*
Cleft palate without cleft lip	3.93	1.44	0.58	1.28	1.96	1.83	2.7	1.83	2.29	3.27	2.16	14	-0.66	-16.79	**
Cleft lip without cleft palate	6.3	5.77	4.9	6.25	4.6	4.77	4.47	3.19	3.54	3.2	4.32	11	-3.1	-49.21	**
Cleft lip with Cleft palate	9.44	8.9	9.51	8.17	9.55	8.01	8.09	7.49	8.4	4.8	7.88	6	-4.64	-49.15	
Congenital microtia	2.89	2.89	2.45	1.79	2.3	1.62	2.51	1.83	2.29	2.37	2.22	13	-0.52	-17.99	*
other malformation of external ear	15.74	20.21	12.1	16.84	16.11	17.14	17.49	20.54	17.25	18.1	17.39	2	2.36	14.99	
Congential esophageal atresia	1.05	0.72	0.72	0.38	0.69	0.71	1.21	1.27	0.74	0.14	0.75	22	-0.91	-86.67	
Congenital atresia of rectum and anus	3.67	2.65	3.31	2.3	2.53	2.23	3.07	2.47	2.36	3.41	2.76	12	-0.26	-7.08	
Hypospadias	4.2	4.81	5.33	4.34	6.33	3.85	6.14	4.86	5.16	6.06	5.23	8	1.86	44.29	
Extrophy of urinary bladder	0	0	0	0	0.23	0	0	0	0	0	0.02	24	0	—	
Congenital talipesequinovarus	6.82	8.18	7.06	6.63	7.36	9.73	12	11.39	8.77	8.77	9.05	5	1.95	28.59	
Polydactyly	12.59	15.88	13.83	14.42	17.83	15.51	14.98	11.47	17.4	17.82	15.43	3	5.23	41.54	
Syndactyly	4.72	6.98	4.75	3.44	4.83	4.36	4.74	4.78	5.53	6.27	5.06	9	1.55	32.84	**
Limp reduction defects	6.82	8.9	4.32	5.49	4.72	5.17	4.56	3.5	3.24	3.97	4.56	10	-2.85	-41.79	*
Congenital diaphragmatic hernia	0.26	0.24	0.72	0.64	0.81	0.81	1.12	0.8	1.18	1.04	0.87	20	0.78	300.00	
Omphalocele	1.31	2.89	1.15	1.15	1.84	0.2	1.21	1.59	1.11	0.97	1.23	19	-0.34	-25.95	**
Gastroschisis	2.36	1.68	2.3	2.17	1.61	2.43	0.93	1.43	0.88	0.84	1.5	16	-1.52	-64.41	
Conjoined twins	0	0.24	0.14	0.26	0.12	0.1	0.09	0.08	0	0.07	0.1	23	0.07	—	
Trisomy 21 syndrome	1.05	1.44	1.15	1.4	1.27	1.42	1.02	1.04	1.25	1.39	1.24	18	0.34	32.38	**
Congenital heart defects	22.56	29.84	21.72	27.43	34.31	52.73	75.63	66.89	59.27	74.00	53.1	1	51.44	228.01	**
Congenital malformation of kidney	7.61	8.42	6.77	7.03	11.85	9.84	13.95	15.13	12.31	14.62	11.7	4	7.01	92.12	**

D_♀_ = (prevalence rate in 2014)-(prevalence rate in 2005); Extent%∆ = D/(prevalence rate in 2005)×100%

* for p<0.05

** for p<0.01

### Associations of prevalence of BDs with maternal characteristics

Totally 444485 perinatal infants in urban areas (48.03%) and 480928 in rural areas (51.97%) were monitored, and the average prevalence rate of BDs in urban areas was significantly higher than that in rural areas (209.66 vs. 175.41 per 10000 PIs) (*OR* = 1.20, 95%CI: 1.16–1.24, *P* = 0.000) (Table 3). There were 491527 male perinatal infants (53.11%),433594 female perinatal infants(46.85%) and 292 with unknown gender (0.03%), and the average prevalence of BDs in male perinatal infants was significantly higher than in female perinatal infants(205.46 vs. 173.27 per 10000 PIs) (*OR* = 1.19, 95%CI: 1.15–1.23, *P* = 0.000). The average prevalence of BDs in the mothers above age 35 was significantly higher than that in mothers under age 35(*OR* = 1.24, 95%CI: 1.17–1.30,*P* = 0.000).

**Table 3:** Prevalence rates of BDs and crude ORs between 2005 and 2014 analyzed from the perspective of maternal characteristics.

	BDs(n)	Perinatal infants(n)(%)	Prevalence of BDs (per 10000 PIs)(95%CI)	OR(95%CI)
**Region**				
urban	9319	444485(48.03)	209.66(208.46–210.86)	1.20(1.16–1.24)
rural	8436	480928(51.97)	175.41(174.31–176.51)	reference
**Infant gender**				
male	10099	491527(53.11)	205.46(204.36–206.56)	1.19(1.15–1.23)
female	7513	433594(46.85)	173.27(172.17–174.37)	reference
**Maternal age**				
<20	256	13535(1.46)	189.14(182.54–195.74)	reference
20~24	5319	282959(30.58)	187.98(186.58–189.38)	
25~29	7358	394663(42.65)	186.44(187.64–185.24)	
30~34	3253	165575(17.89)	196.47(194.57–198.37)	
35~	1568	68681(7.42)	228.3(225.15–231.45)	1.24(1.17–1.30)

## Discussion

**Fig 1:**
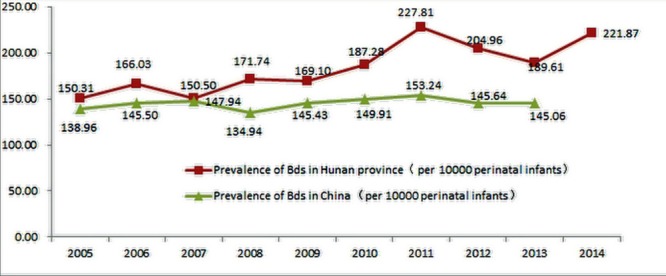
The trends of BDs prevalence rates in Hunan and in China from 2005 to 2014.

The prevalence rate of total BDs in Hunan increased by 4.76% annually from 2005 to 2014, which is more significant than that at the state level, which increased by3.97% annually from 2005 to2013 (Fig 1). In addition, the prevalence rate of BDs in Hunan was higher than that of China, and the main five types were CHD, CMK, polydactyly, OMEE, and CTE. Moreover, the prevalence rates of BDs were higher in urban perinatal infants versus rural, higher in male perinatal infants versus female and higher in mothers above age 35 versus mothers under age 35.

The total prevalence rates of BDs in Hunan show upward trend and the main five BDs were CHD, OMEE, polydactyly, CMK, and CTE in the last 10 years. First, this phenomenon might be attributed to the higher-level prenatal diagnosis including better and more accessible ultrasonography, genetics techniques, biological immune technology and so on, which allows the identification of potential cases especially minor anomalies. Such as, better and more accessible perinatal ECHO cardiography services could increasing CHD to some extent. Second, the occurrence of BDs would be attributed to the individual genetic background and environment determinants[13]. Third, with the increased awareness and improved reporting over the decade, the underreporting errors of BDs were reduced.

From 2005 to 2014, the prevalence rates of congenital hydrocephalus, NTDs, cleft lip with cleft palate, cleft lip without cleft palate, and limb reduction defects in Hunan all decreased. The prior option is often termination of pregnancy in China to these severe BDs[14, 15]. This phenomenon might be attributed to the innovation and popularization of prenatal diagnosis technology, which lead the prenatal diagnosis of time bring forward. An increasing number of BDs (especially, severe BDs were diagnosed at less than 28 weeks), were selective termination of pregnancy[8], and they are not included in the calculation of BDs prevalence rates if less than 28 weeks. Moreover, NTDs were confirmed to be associated with the peri-conceptional folic acid deficiency[16, 17]. Since the Chinese government promoted folic acid supplement[18], the prevalence rates of NTDs in China and in Hunan continued to decline in the past 10 years.

The average prevalence of BDs in urban Hunan is significantly higher than that in rural Hunan, which is similar to the Chinese Birth Defect Surveillance System with higher prevalence of BDs in urban areas than in rural areas especially CHD from 2007[19–20]. The phenomenon is explained to some extent by the stronger overall health awareness, the wider use of prenatal diagnosis techniques, more accessibility and reporting practices in urban areas. We also find the prevalence rate of BDs is significantly higher in male versus female perinatal infants, which is consistent with some previous studies [20–22]. In a recent population-based study in the United Kingdom, the overall risk of congenital anomalies in males is greater than in females, which might be explained by the higher susceptibility of Y chromosome than X chromosome[23]. Besides, the external genital deformities in males are more detectable than in females[24,25]. Some previous studies have reported the impacts of maternal age on BDs. Our study shows that pregnant women above age 35 are more likely to give birth with BDs. As reported, advanced maternal age (35–40 years old) is associated with CHD in infants(OR = 1.12,95%CI: 1.03–1.22),and young maternal age (14–19 years old) has a significant high risk of cleft lip in infants(OR = 1.88, 95%CI: 1.30–2.73)[26]. Moreover, the increased risks of cleft lip with or without palate in mothers above age 35 (adjusted OR = 2.12, 95% CI: 1.26–3.57),and young maternal age<25 are associated with a reduced risk of CHD (adjusted OR = 0.73, 95%CI: 0.59–0.90) and an elevated risk of polydactyly (adjusted OR = 1.42, 95%CI: 1.09–1.84) in infants[27]. The discrepancy above may be attributed to the smallest population of only 256 perinatal infants with BDs with maternal age younger than 20 in our study, which was too small to reveal any statistical significance. Meanwhile, pregnant women under age 20 are unmarried and often choose the termination of pregnancy before 28 weeks of gestation. Thus, the impacts of young maternal age (< 20 years old) on BDs deserve further investigation.

This study illustrates the epidemiological characteristics of BDs in the most populated province, Hunan, for the first time based on a long-term dynamic Birth Defect Surveillance System in China. Our study elaborates the trends of 24 types of BDs. However, there are some limitations. First, there is no individual database for each pregnancy, so we cannot analyze the determinants such as multiple births, parity, smoking, drinking alcohol, socioeconomic status, medical history, prenatal care, or paternal details. Second, we did not have information regarding termination at less than 28 weeks of gestation in women with severe congenital anomalies, which could under estimate the calculated total prevalence of BDs especially certain major prenatally diagnosed defects. The described trends of BDs only represent perinatal infants. Third, the most common and increasing prevalences especially CHD, OMEE and polydactyly are affected to some extent by definition criteria, increasing surveillance, reporting and changes in diagnostic techniques and their availability. On the other hand, the prevalence changes of major defects (congenital hydrocephalus, NTDs, limb reduction) which are not so susceptible to changes in data collection methods showed a decreasing prevalence. The difference in accessibility and reporting practices between rural and urban women be leading to different prevalence rates in rural vs urban women to some extent. However, The affection of definition criteria, and improved reporting, the progress of diagnostic techniques, increased awareness, the difference in accessibility to the prevalence rate of BDs could not be separated from the total change of BDs. In summary, the rate of total BDs in perinatal infants in Hunan has increased in the last 10 years. CHD, OMEE, polydactyly, CMK, and CTE increased much and congenital hydrocephalus, NTDs declined much. Urban pregnant women, male perinatal infants, and mothers above age 35 present different prevalence rates of BDs. New diagnosis technology, improve the ability of monitoring, strengthen the publicity and education are important to decline the prevalence of BDs.
